# The Relative Age Effect across an International Soccer Programme in Male and Female Players Aged 12 Years Old to Seniors

**DOI:** 10.5114/jhk/186563

**Published:** 2024-07-17

**Authors:** Ryland Morgans, John Radnor, Daniel Nisbet, Jose Teixeira, Toni Modric, Eduard Bezuglov, Halil İbrahim Ceylan, Ronan Kavanagh, Piotr Zmijewski, Rafael Oliveira

**Affiliations:** 1 School of Sport and Health Sciences, Cardiff Metropolitan University, Cardiff, UK.; 2 FA of Wales, Hensol, Cardiff, UK.; 3 SPRINT—Sport Physical activity and health Research and Innovation Center, Guarda, Portugal.; 4 LiveWell Research Centre for Active Living and Wellbeing, Polytechnic Institute of Bragança, Bragança, Portugal.; 5 Faculty of Kinesiology, University of Split, Split, Croatia.; 6 High Performance Sport Center, Croatian Olympic Committee, Zagreb, Croatia.; 7 Department of Sports Medicine and Medical Rehabilitation, Sechenov First Moscow State Medical University, Moscow, Russia.; 8 Physical Education of Sports Teaching Department, Faculty of Kazim Karabekir Education, Ataturk University, Erzurum, Turkey.; 9 Football Performance Hub, University of Central Lancashire, Preston, UK.; 10 Faculty of Physical Education, Jozef Pilsudski University of Physical Education in Warsaw, Warsaw, Poland.; 11 Research Center in Sport Sciences, Health Sciences and Human Development (CIDESD), Santarém Polytechnic University, Rio Maior, Portugal.; 12 Santarém Polytechnic University, School of Sport, Rio Maior, Portugal.

**Keywords:** youth players, senior players, soccer, international teams, relative age effect

## Abstract

The purpose of the study was to examine the prevalence of the Relative Age Effect (RAE) across an international soccer programme in male and female players aged 12 years old to seniors. One hundred forty-five male (age: 18.8 ± 4.6 years; body mass: 68.1 ± 10.2 kg; body height: 177.3 ± 10.5 cm) and 218 female (age: 15.9 ± 4.6 years; body mass: 66.2 ± 10.5 kg; body height: 170.6 ± 8.3 cm) players from a National Association were assessed. All participants were divided into four quartiles: January to March (BQ1), April to June (BQ2), July to September (BQ3), and October to December (BQ4). The results showed that the distributions for all male squads were significantly skewed, with more players than expected from BQ1 in the U-21, U-19, and U-17 and less players than expected from BQ4 in the U-19 squad. The distributions for all female squads showed significantly more players than expected from BQ1 in the U-16 and less players than expected from BQ4 in the U-14 squad. The distributions across the different positions for the male squads combined were significantly skewed with more forward players than expected from BQ1 and less forward and midfielder players than expected from BQ4. For the female squads, there were significantly more BQ1 defenders from the U-16 squad than expected. In conclusion, this study unveils significant disparities in quartile distributions among male and female squads. Moreover, the data emphasize the potential impact of heightened physical demands in certain positions on the RAE.

## Introduction

The Relative Age Effect (RAE) is considered widespread in various sports (Cobley et al., 2009; Romann et al., 2018) and has been particularly highlighted in young male athletes aged 15 to 18 years old, including soccer and basketball players (Brustio et al., 2019). January 1 ^st^ is the commencement of the sporting calendar year and is often employed to categorize players into specific age groups for training and competitive match-play (Bezuglov et al., 2022), although in elite soccer the competitive season is from August through to May. Children born closer to the cut-off date are more likely to be cognitively, physically and psychologically more developed than children born further away from the cut-off date (Cobley et al., 2009; Malina et al., 2015). This phenomenon provides chronologically older athletes with an immediate competitive advantage over younger counterparts (Figueiredo et al., 2019).

Notably previous research examining the RAE highlighted that lower overall performance levels and, potentially drop-out prior to attaining and maximizing full potential may occur in athletes born further away from the cut-off date (Hollings et al., 2014). Recent research has identified the RAE in younger age groups (6 to 12 years old) in hockey, soccer, and alpine skiing (Bezuglov et al., 2019; Lemoyne et al., 2021). In the early stages of development, chronologically older athletes are biologically more mature, that results in physical and cognitive gains (Bezuglov et al., 2022), while the occurrence of the RAE in athletes aged 15 to 18 years is most evident in elite male athletes (Jakobsson et al., 2021). Of note is the scant data focused on female athletes (Brustio et al., 2023). The occurrence of the RAE among elite adolescent athletes in these sports could be attributed to older peers who, being chronologically advanced, are more prone to entering puberty earlier. Consequently, these athletes may gain advantages in strength, speed, endurance, and bone strength owing to the impact of testosterone (Handelsman et al., 2018). Therefore, late-born children who mature early may not be disqualified from highly competitive sports, as observed by some authors, given that the number of late-maturing children diminishes significantly with an increase in chronological age (Figueiredo et al., 2009).

The RAE in soccer is considered prevalent and has previously been described as an over-representation of athletes born in the period from January to March, while there is a lower representation of athletes born in October and November when considering age group classifications (Ibáñez et al., 2018; Perez-Gonzalez et al., 2020). Earlier work by Williams et al. (2010) that examined a sample of players from the men’s FIFA U-17 World Cup reported that approximately 40% were born between January and March, while only 16% were born between October and December. Furthermore, Rada et al. (2018) identified a significant RAE in five European male soccer leagues and the corresponding second-tier competitions. The research revealed a two-fold difference in the number of players born in the first month of the calendar year compared to those born in the last month, indicating a persistent RAE in both top-tier and second-tier leagues. Thus, it seems evident that no second chances were available for later born players in that study (Rada et al., 2018). Therefore, diminishing the likelihood of recovering talented players dismissed from youth programs is solely based on delayed maturity. More recently the RAE has proven prevalent in elite German (Götze and Hoppe, 2020) and Scottish (Dugdale et al., 2021) male soccer players, however, in specific European soccer groups, the presence of the RAE was not apparent among older players in comparison to younger counterparts (Bezuglov et al., 2019). Recently, it was highlighted that approximately 17% of players selected for youth teams progressed to the national senior team, signifying a substantial turnover in Italian youth squads, along with the observation of the RAE in both U-17 and U-19 categories (Brustio et al., 2023). In addition, the same study did not find any differences among quartiles in transition rates in the Italian female soccer context (Brustio et al., 2023).

In team sports like soccer, the common acknowledgment of the RAE during adolescence may be attributed to the very early selection process in both male and female soccer associations. This assessment typically occurs between the age of 6 and 8 years old, contributing to the primary over-representation of early-born players, a trend that may persist until late puberty (Bezuglov et al., 2022). Despite the wealth of research on the RAE in various contexts, there is limited investigation into its prevalence in elite youth female soccer groups within the contemporary scientific literature (Brustio et al., 2023). Therefore, this study aimed to fill this gap by investigating the prevalence of the RAE in elite male and female national players across various age groups, ranging from 12 years old to seniors. Building on previous research involving U-16 to U-23 players (Brustio et al., 2023), the study hypothesis was that the RAE would be evident across all elite age groups within the national team training program.

## Methods

### Participants

One hundred and forty-five male (mean ± SD, age: 18.8 ± 4.6 years; body mass: 68.1 ± 10.2 kg; body height: 177.3 ± 10.5 cm) and 218 female (mean ± SD, age: 15.9 ± 4.6 years; body mass: 66.2 ± 10.5 kg; body height: 170.6 ± 8.3 cm) soccer players from a national association were assessed. The sample players were based on national team selection at specific age groups. The study cohort consisted of male international players classified into age groups: U-15 (n = 24), U-16 (n = 26), U-17 (n = 22), U-19 (n = 23), U-21 (n = 24), and senior (n = 26). Female international players were classified into the following age groups: U-15 (n = 27), U-17 (n = 20), U-19 (n = 23), and senior (n = 24) and regional players were classified into: U-12 (n = 40), U-14 (n = 24), U-16 (n = 36), and U-18 (n = 24). The number of participants in each age group varied from 20 to 36 and this was determined by players that competed at international and regional levels during the 2023/2024 season. Participants were also classified into four groups based on playing position: goalkeepers (GK, male: n = 13, female: n = 24), defenders (DF, male: n = 38, female: n = 65), midfielders (MF, male: n = 39, female: n = 95), and forwards (FW, male: n = 28, female: n = 33).

All participants were further divided into four quartiles according to the month of birth: January to March, first quartile (BQ1, "early-born"); April to June, second quartile (BQ2); July to September, third quartile (BQ3); and October to December, fourth quartile (BQ4, "late-born"). This method of division has previously been validated in various athletes (Bezuglov et al., 2022; Brazo-Sayavera et al., 2018) and soccer players (Figueiredo et al., 2019). The occurrence of the RAE was analyzed across all national players, born in various quarterly periods, and across each age group (male: U-15 to seniors, female: U-12 to seniors).

### Measures

Two independent experts examined the birthdate information of the national association squads. All compiled data resulted from standard analytical procedures related to players’ monitoring throughout the competitive calendar; however, written consent was provided by all participants, and for those under 18 years old, consent was obtained from parents or guardians. The study adhered to the principles of the Declaration of Helsinki and received approval from the local Ethics Committee of the University of Central Lancashire (approval code: BAHSS 646; approval date: 17 April 2019) and the relevant National Association, where the participants volunteered (Winter and Maughan 2009). To maintain confidentiality, all data underwent anonymization before analysis.

### Statistical Analysis

Frequency counts were employed to ascertain players’ distribution across each birth quartile (BQ1–4) in both the male and female national squads. Additionally, the calculation of players from each birth quartile for various positions was conducted. To assess the birth quartile distribution, a chi-square (χ2) analysis was applied to compare the observed sample with the expected distribution based on population values (Office for National Statistics, 2015).

The following equation was used to calculate the chi-square (χ2) value:


$$\chi^{2}=\sum \frac{\left(observed-model\right)2}{model}$$

In this scenario, the model values were computed based on national norms, with percentages set at 25.5% for BQ1, 24.5% for BQ2, 24.7% for BQ3, and 25.4% for BQ4 (Office for National Statistics, 2015). The discrepancy between the observed player count in each birth quartile and the expected count according to national norms was squared, divided by the expected number, and summed to obtain the chi-squared value. As the χ2 test alone does not convey the extent of difference in birth quartile distributions for statistically significant output, Cramer’s *V* was utilized to gauge the magnitude of difference in frequency counts. The interpretation of Cramer’s *V* involved categorizing values from 0.06 to 0.16 as a small effect size, 0.17 to 0.28 as a medium effect size, and >0.29 as a large effect size (Cohen, 1988). Additionally, analysis of the adjusted standardized residuals was conducted to pinpoint frequencies exceeding 1.96 or falling below −1.96 z-scores (*p* < 0.05), indicating a noteworthy deviation from the expected distribution for each age group.

## Results

**Figure 1:**
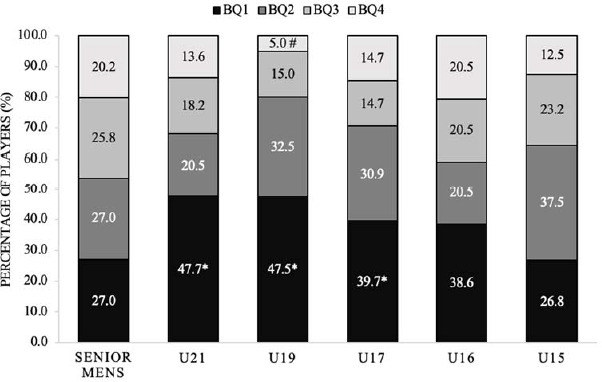
Percentage of players within each birth quartile for all male squads. BQ: birth quartile; * significantly more than expected (p < 0.05); # significantly less than expected (p < 0.05)

The distribution of birth quartiles in all male squads exhibited significant skewness with a moderate effect size compared to the normal distribution (χ2 (*df* = 3) = 33.9, *p* < 0.05, *V* = 0.182) (Figure 1). The adjusted residuals indicated a significant over-representation of players from BQ1 in the U-21, U-19, and U-17 age groups, as well as a significant under-representation of players from BQ4 in the U-19 squad (*p* < 0.05).

**Figure 2:**
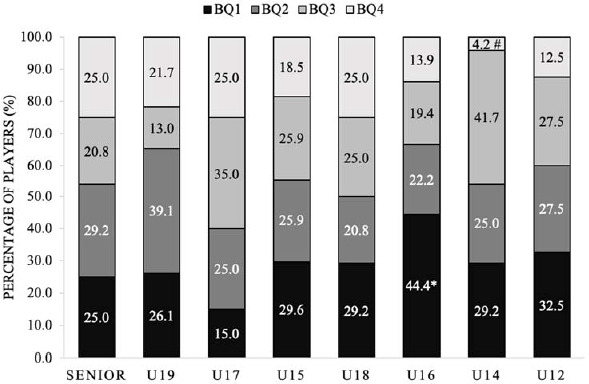
Percentage of players within each birth quartile for all female squads. BQ: birth quartile; * significantly more than expected (p < 0.05); # significantly less than expected (p < 0.05)

The distribution of birth quartiles across all female squads did not differ significantly from expected values, demonstrating a small effect size compared to the normal distribution (χ2 (*df* = 3) = 7.96, *p* = 0.25, *V* = 0.110) (Figure 2). The adjusted residuals indicated a significant over-representation of players from BQ1 in the U-16 age groups and a significant under-representation of players from BQ4 in the U-14 squad (*p* < 0.05).

**Figure 3:**
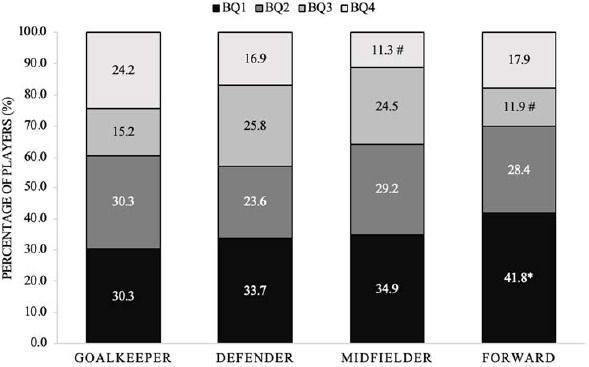
Percentage of players in each position from specific birth quartiles for all male squads combined. BQ: birth quartile; * significantly more than expected (p < 0.05); # significantly less than expected (p < 0.05)

The distribution of birth quartiles across various positions for the combined male squads exhibited significant skewness with a small effect size compared to the normal distribution (χ2 (*df* = 3) = 25.00, *p* < 0.05, *V* = 0.168) (Figure 3). The adjusted residuals revealed a significant over-representation of forward players from BQ1 and a significant under-representation of forward players from BQ3, as well as midfielders from BQ4 (*p* < 0.05).

**Figure 4:**
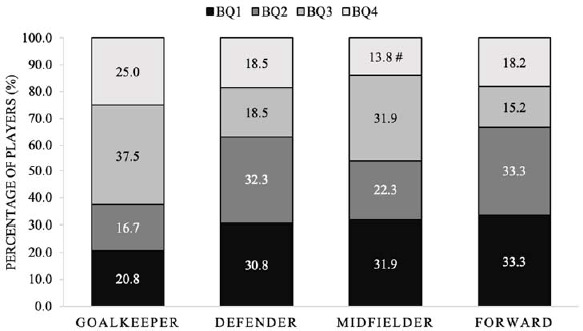
Percentage of players in each position from specific birth quartiles for all female squads combined. BQ: birth quartile; # significantly less than expected (p < 0.05)

The distribution of birth quartiles across various positions for the combined female squads displayed significant skewness with a small effect size compared to the normal distribution (χ2 (*df* = 3) = 8.51, *p* < 0.05, *V* = 0.115) (Figure 4). The adjusted residuals indicated a significant under-representation of midfielders from BQ4 (*p* < 0.05).

Birth quartile frequency counts for each position across the individual male squads are presented in Table 1. There was no difference in the number of players from each birth quartile across positions considering what was expected for the senior, U-21, U-16, and U-15 squads (χ2 (*df* = 3) = 1.30–12.01, *p* = 0.06–0.60, *V* = 0.070–0.302). For the U-19 and U-17 squads, the birth quartile distribution for specific positions was significantly skewed with a medium to large effect size compared to normal distribution (χ2 (*df* = 3) = 6.00–15.49, *p* = 0.016–0.010, *V* = 0.186–0.364). The adjusted residuals highlighted that there were significantly more BQ1 goalkeepers from the U-21 squad, midfielders from the U19 squad, and forwards from the U-17 squad than expected. There were also more goalkeepers from BQ2 in the U-15 squad than expected.

**Table 1:** Frequency count of male players based on the birth quartile and the playing position in each squad.

	Goalkeeper				Defender				Midfielder				Forward			
	BQ1	BQ2	BQ3	BQ4	BQ1	BQ2	BQ3	BQ4	BQ1	BQ2	BQ3	BQ4	BQ1	BQ2	BQ3	BQ4
Senior Male	2	1	1	3	6	5	9	6	10	10	9	5	6	8	4	4
U21	4*	1	1	0	4	3	2	2	9	3	5	2	4	2	0	2
U19	1	3	0	1	7	4	3	0	7*	3	1	1	3	3	2	0
U17	1	1	2	3	7	5	3	2	5	9	5	2	8*	2	0	3
U16	2	1	1	0	5	2	5	3	3	2	3	2	4	2	0	2
U15	0	3*	0	1	1	2	1	2	3	4	3	0	3	2	2	1

BQ: birth quartile; * significantly more than expected (p < 0.05)

For the female squads, birth quartile frequency counts for each position across the individual squads are presented in Table 2. There was no difference in the number of players from each birth quartile across positions compared to what was expected for all squads (χ2 (*df* = 3) = 0.11–7.22, *p* = 0.20–0.70, *V* = 0.040–0.317) except for the U-16 squad (χ2 (*df* = 3) = 7.45, *p* = 0.05, *V* = 0.263). The adjusted residuals highlighted that there were significantly more BQ1 defenders in the U-16 squad than expected.

**Table 2:** Frequency count for female players based on birth quartile and playing position in each squad.

	Goalkeeper				Defender				Midfielder				Forward			
	BQ1	BQ2	BQ3	BQ4	BQ1	BQ2	BQ3	BQ4	BQ1	BQ2	BQ3	BQ4	BQ1	BQ2	BQ3	BQ4
Senior Female	1	0	0	2	1	1	0	3	4	4	4	1	0	1	1	0
U19	2	0	1	1	2	5	0	3	1	3	2	1	1	1	0	0
U17	0	0	2	0	0	3	1	2	2	2	3	2	1	0	1	0
U15	1	0	1	1	1	2	1	1	4	3	5	1	2	2	0	2
U18	0	1	0	1	3	3	2	1	4	0	4	3	0	1	0	1
U16	0	2	1	1	7*	2	1	2	6	2	5	1	3	2	0	1
U14	1	0	2	0	3	4	4	0	2	1	4	1	1	1	0	0
U12	0	1	2	0	3	1	3	0	7	6	3	3	3	3	3	2

BQ: birth quartile; * significantly more than expected (p < 0.05)

## Discussion

The main findings of this study reveal notable variations in the birth quartile distribution between male and female international youth soccer teams. The birth quartile distributions for all male squads were significantly skewed, with significantly more players than expected from BQ1 in the U-21, U-19, and U-17 age groups and significantly less players than expected from BQ4 in the U-19 squad. Considering all female squads, the birth quartile distributions were non-significantly different compared to expected values with significantly more players than expected from BQ1 in the U-16 age groups and significantly less players than expected from BQ4 in the U-14 squad. The birth quartile distributions across the different positions for the male squads combined were skewed with significantly more forward players than expected from BQ1 and significantly less forward and midfielder players than expected from BQ4. Moreover, there were no disparities in the count of players from each birth quartile across positions. However, in the U-19 and U-17 squads, the birth quartile distribution for certain positions exhibited significant skewness, with notably more BQ1 goalkeepers in the U-21 squad, midfielders in the U-19 squad, and forwards in the U-17 squad than anticipated. There were also more goalkeepers from BQ2 in the U-15 squad than expected. For the female squads, birth quartile frequency counts for each position across the individual squads presented no difference in the number of players from each birth quartile across positions except for the U-16 squad with significantly more BQ1 defenders from the U-16 squad than expected.

Regarding the male squads, the examination of adjusted residuals revealed a significantly greater number of players from BQ1 in the U-21, U-19, and U-17 age groups. This suggests that an elevated percentage of players in these age groups were born in the initial months of the year. Additionally, there were significantly fewer players than expected from BQ4 in the U-19 squad, indicating that there is a lower proportion of players born in the later months of the year in this squad. The present data align with earlier findings in men's international soccer, as evidenced by Işın (2021) who reported that 40.6% of players across FIFA U-17 and U-20 World Cups from 2009/2019 were born in BQ1, whereas only 16.9% were born in BQ4. Similarly, Williams (2010) determined that 40% of players across all participating countries in the U-17 World Cup between 1997 and 2007 were born in BQ1, while only 16% were born in BQ4.

In particular, the absence of a significant difference for the male U-15 and U-16 squads, contradicts previous research in youth soccer (Bezuglov et al., 2019; Sweeney et al., 2022). This incongruity may be attributed to the non-tournament structure of these two age groups, where a heightened emphasis on long-term development is placed. The effective understanding of the RAE may mitigate potential biases associated with birth quarter effects, thus contributing to the observed lack of significant disparities in players’ representation across different birth quartiles.

Similarly, for the female squads, the analysis of adjusted residuals indicated a notable increase in the number of players born in BQ1 in the U-16 age group. This implies that a greater percentage of players in the U-16 squad were born in the initial months of the year. Moreover, there were notably fewer players than anticipated from BQ4 in the U-14 squad, signifying a reduced proportion of players born in the later months of the year within this squad. The findings in these two age groups substantiate earlier research examining international female data for the U-17 and U-19 categories (Brustio et al., 2023), revealing a notable 35% representation of players born in BQ1 and a comparatively lower 18% representation of players born in BQ4. It is pertinent to note that the previous study lacks data representing U-14 and U-16 cohorts.

The research by Brustio et al. (2023) postulates the potential existence of a higher rate of progression from youth to senior teams among players born in later birth quartiles, aligning with the underdog hypothesis. The non-significant differences identified within the U-12, U-15, U-17, and U-19 teams, while not immediately impactful, may bear longer-term benefits for the national setup. The absence of significant disparities in these younger age groups implies a potential equilibrium in talent development across birth quartiles, which could contribute to a more balanced and sustainable talent pool for the senior national team over time.

When examining the birth quartile distribution across different playing positions for the male squads combined, there were significant differences compared to a normal distribution. Specifically, there were significantly more forward players than expected from BQ1, suggesting that a higher percentage of forward players were born in the earlier months of the year. Moreover, there was a notable scarcity of forwards and midfielders compared to the anticipated numbers from BQ4, signifying a decreased proportion of players born in the later months of the year in these specific positions.

The association between physical development and athletic performance is well-established in the literature, with more physically developed players exhibiting superior attributes such as size, strength, speed, power, agility, and endurance (Meylan et al., 2010). Moreover, Parr et al. (2021) highlight that these players manifest higher in-game physical prowess, attaining greater speed, covering increased distances at high speed, and executing more accelerations. This heightened physical capacity translates into greater involvement in both attacking and defensive actions (Johnson, 2022). Consequently, the pre-disposition to select more physically developed players in positions demanding heightened challenges, such as central defenders and forwards, is a logical outcome.

The identification of significantly fewer midfielders from BQ4 supports previous research (Işın, 2021). However, it is imperative to acknowledge a limitation within the current study, wherein only four playing position groups were considered. The absence of differentiation between midfielders to that of defensive, central, attacking, and wide precludes a comprehensive understanding of potential variations among these roles. Işın (2021) proposed that later maturing and physically less developed players were frequently deployed in more technical midfield positions (attacking), emphasizing technical ability over physical attributes. It is noteworthy, however, that the present research fails to substantiate these assertions, highlighting the need for future investigations to delve into the nuanced interplay between physical development, playing positions, and technical demands in soccer.

Similarly, the birth quartile distribution across different positions for the female squads combined was significantly skewed compared to a normal distribution. There were significantly fewer midfielders than expected from BQ4, suggesting that there was a lower proportion of midfielders born in the later months of the year. The present dataset is reflective of antecedent investigations in female international soccer (Brustio et al., 2023), wherein discernible asymmetries in relative age were observed within defenders and midfielders at U-17 level, indicating a medium effect size. Notably, the examination of playing positions has elucidated an augmentation of the RAE, particularly notable among female goalkeepers and midfielders in Spain (Sedano et al., 2015), and the United States (Baker et al., 2009). Similar trends were identified among defenders, midfielders, and forwards from Switzerland (Romann and Fuchslocher, 2011), and exclusively within midfielders when scrutinizing Women’s Soccer World Cup rosters (U-17 and U-19) (Ribeiro et al., 2023). While the specific causal mechanisms underlying these observed trends have yet to be conclusively determined, it is posited that the heightened physical demands intrinsic to certain playing positions (Schorer et al., 2009) may partly explain the emergence of the observed RAE. In the present study, there were no differences in the number of players from each birth quartile across positions, except in the U-19 and U-17 squads. In these squads, the birth quartile distribution for specific positions was significantly skewed compared to a normal distribution. Notably, there were significantly more BQ1 goalkeepers in the U-21 squad, midfielders in the U-19 squad, and forwards in the U-17 squad than expected. Additionally, there were more goalkeepers from BQ2 in the U-15 squad than expected.

The positional demands and performance advantages associated with goalkeepers possessing greater stature and heightened power levels provide justification for the pre-disposition toward a higher representation of goalkeepers in BQ1. This observation is consistent with earlier research by Del Campo (2010). Furthermore, Işın (2021) postulated that in positions like defenders and goalkeepers, where physical characteristics play a predominant role, the manifestation of this effect may be more pronounced.

Table 2 presents the birth quartile frequency counts for each position across the individual female squads. In general, there were no variations in the players count from each birth quartile among positions, except for the U-16 squad. In this squad, there were significantly more BQ1 defenders than expected. This is in contrast with previous research that found the RAE across all age groups measured (Brustio et al., 2023; Ribeiro et al., 2023). The findings in the U-16 squad are consistent with previous research (Romann and Fuchslocher, 2011) and support the hypothesis that there is a bias to earlier born players in positions of heightened physical demands (Schorer et al., 2009). This further demonstrates the variable nature within women’s soccer which may become more apparent with increased research.

## Limitations

Several limitations warrant consideration when interpreting the findings of this study. Firstly, only birth dates were recorded, omitting essential anthropometric data, maturation status, and performance variables crucial for a comprehensive understanding of physical status and the RAE. This omission may constrain the depth of our analysis. Secondly, the methodology involved considering all players selected within specific age groups at a given point in time, without accounting for the frequency of selections. This oversight may neglect potential fluctuations in players’ selection and progression through international age groups over time, potentially impacting the interpretation of results. Furthermore, the study indiscriminately included both friendly and official matches, without distinguishing between the different types of matches. This lack of differentiation may overlook nuances that could arise in different match contexts, influencing the generalizability of the present findings. A further notable limitation pertains to the classification of playing positions, which was confined to four broad categories (GK, CD, CM, FW). This over-simplified classification ignores substantial differences in the physical demands placed on players in wide positions (Di Salvo et al., 2007; Richard and Tschopp, 2012). As proposed by Martines-Lagunas (2014), a more nuanced classification involving six categories (GK, CD, WD, CM, WM, FW) could potentially reveal more insightful findings related to selection bias, particularly in understanding how physical demands vary across different playing positions during both men's and women's match-play.

## Conclusions

In summary, this research reveals notable discrepancies in birth quartile distributions between male and female national soccer squads. Male squads show a biased distribution, particularly favoring players born in BQ1 across different age groups, while female squads demonstrate distributions closer to the expected values. The over-representation of players born in the earlier months of the year in various squads suggests potential implications for talent identification and developmental pathways within the national soccer program. Analysis of birth quartile distributions across playing positions identifies significant deviations for male squads, particularly in the forward position, supporting the established link between physical development and players’ selection. Similarly, female squads exhibit skewed birth quartile distributions across positions, with fewer midfielders than expected from BQ4. The data reflect asymmetries in relative age observed in previous research, emphasizing the potential impact of heightened physical demands in certain positions. These findings emphasize the importance of further examining the impact of birth month effects in soccer and other sports. Such considerations are crucial, as these factors may potentially contribute to biases in players’ selection and developmental processes.
